# Genetic Variants in Antioxidant Genes Modulate the Relationships Among Obesity-Related Oxidative Stress Markers in Mexican Children

**DOI:** 10.3390/antiox14080896

**Published:** 2025-07-22

**Authors:** Ana Nava-Cabrera, Armando Ramírez-Cruz, Jaime Gómez-Zamudio, Araceli Pérez-Bautista, Linda Esther Ruiz-Queb, Miguel Vazquez-Moreno, Miguel Cruz

**Affiliations:** 1 Unidad de Investigación Médica en Bioquímica, Hospital de Especialidades, Centro Médico Nacional Siglo XXI, Instituto Mexicano del Seguro Social, Av. Cuauhtémoc 330, Col. Doctores, Delegación Cuauhtémoc, Mexico City C.P. 06720, Mexico; annetmx.qfb@gmail.com (A.N.-C.); armando_050688@hotmail.com (A.R.-C.); moreno_029@yahoo.com.mx (M.V.-M.); 2 Programa de Doctorado en Ciencias Médicas, Odontológicas y de la Salud, Universidad Nacional Autónoma de México, Mexico City C.P. 04510, Mexico; 3 OOAD DF Norte del Instituto Mexicano del Seguro Social, Unidad de Medicina Familiar No. 23, Mexico City C.P. 07450, Mexico; 4 OOAD Campeche del Instituto Mexicano del Seguro Social, Unidad de Medicina Familiar No. 13. Cd. Concordia, Campeche C.P. 24085, Mexico

**Keywords:** childhood obesity, oxidative stress, antioxidant genes, single-nucleotide polymorphisms, lipid peroxidation, Mexico

## Abstract

Single-nucleotide polymorphisms (SNPs) in antioxidant genes could influence redox regulation from early life. We aimed to assess the direct and modulatory effects of SNPs in antioxidant genes (*SOD2* rs4880, *GPX1* rs1050450, *GPX7* rs835337, *CAT* rs1001179) on the relationships among obesity-related oxidative stress markers in Mexican children. Anthropometric data of 2946 unrelated children were analyzed in this cross-sectional study. SNPs were genotyped using TaqMan assay. Serum total antioxidant capacity (sTAC) and oxidative stress markers (thiobarbituric acid reactive substances [TBARS, as lipid peroxidation], and protein carbonyl [PC]) were assessed. Although no SNPs were associated with obesity (*p* ≥ 0.125), both sTAC (*p* = 0.001) and TBARS (*p* = 0.015) were positively associated with it. A negative relationship was also observed between sTAC and TBARS (*p* < 0.001). *SOD2* rs4880 was negatively associated with TBARS, while *GPX1* rs1050450 was inversely associated with both TBARS and PC levels (*p* ≤ 0.036). The inverse association between sTAC and TBARS remained significant only in non-carriers of *SOD2* rs4880 (*p* = 0.003) and *GPX1* rs1050450 (*p* = 0.002). Our data evidence that sTAC and TBARS are associated with obesity, showing a negative relationship in Mexican children who are non-carriers of *SOD2* rs4880 and *GPX1* rs1050450.

## 1. Introduction

Childhood obesity is a serious public health concern with profound metabolic consequences, as it is strongly associated with the risk of early development of metabolic syndrome, non-alcoholic fatty liver disease, dyslipidemia, cardiovascular disease, and type 2 diabetes. In Mexico, the National Health and Nutrition Survey (ENSANUT Continua 2020–2023) reported a combined prevalence of overweight and obesity of 36.5% in schoolchildren aged 6 to 11 [1].

One of the key pathophysiological characteristics of obesity is oxidative stress (OS), which is present when the production of reactive oxygen species (ROS) overtakes the antioxidant defense systems [2]. OS is responsible for oxidative damage to lipids and proteins and is also associated with the development of metabolic complications [3,4].

In the OS, double bonds of polyunsaturated fatty acids in membranes are mostly vulnerable to hydroxyl radical (^•^OH^−^), which extract electrons from them, producing lipid peroxidation products such as aldehydes: malondialdehyde (MDA), 4-hydroxy trans-2-nonenal (HNE), and 4-oxo-2-(E)-nonenal (ONE) [5,6]. Additionally, these lipid aldehydes can subsequently form covalent adducts through their interaction with lysine, histidine, and cysteine residues in proteins, in a process known as protein carbonylation [5,7]. As a result, lipid peroxidation disrupts membranes, affecting cellular signaling and transport, while protein carbonylation promotes loss of enzymatic activity and susceptibility to proteolytic degradation.

The antioxidant systems (enzymatic and non-enzymatic) comprise preventive, neutralizing, and repair mechanisms that collectively maintain cellular redox balance. The enzymatic antioxidant system includes superoxide dismutases (SOD), glutathione peroxidases (GPx), and catalase (CAT) [3]. The non-enzymatic antioxidant system is integrated by exogenous compounds obtained from the diet (vitamins C, E, and A, carotenoids, and polyphenols) as well as endogenous molecules, like reduced glutathione (GSH), uric acid, bilirubin, and albumin [8].

While the effectiveness of the non-enzymatic antioxidant system depends on exogenous factors such as dietary intake, the enzymatic antioxidant defense is influenced by genetic background. In this context, single-nucleotide polymorphisms (SNPs) in antioxidant enzyme genes, such as *SOD2* rs4880, *GPX7* rs835337, *GPX1* rs1050450, and *CAT* rs1001179, have been identified in coding and regulatory untranslated regions of the gene [9,10,11], affecting the net activity of their corresponding enzymes, which compromise the capacity to maintain redox balance. In previous studies, the SNPs *SOD2* rs4880, *GPX7* rs835337, *GPX1* rs1050450, and *CAT* rs1001179 have been associated with lipid peroxidation, obesity, and their metabolic complications in adult populations [10,12,13,14,15]. However, the evidence in pediatric populations remains limited and inconsistent.

Due to the high prevalence of childhood obesity in Mexico and its association with oxidative stress, it is important to investigate how genetic factors related to antioxidant defense impact this condition in pediatric populations. Therefore, the present study aims to investigate both the direct and modulatory effects of four SNPs in antioxidant genes (*SOD2* rs4880, *GPX1* rs1050450, *GPX7* rs835337, *CAT* rs1001179) on the relationships among obesity-related oxidative stress markers in Mexican children.

## 2. Materials and Methods

### 2.1. Study Population

A cross-sectional association study was carried out in 2946 unrelated children of both sexes (1675 with normal weight and 1271 with obesity) aged 6 to 12 years. Participants were recruited through collaboration with local public schools and the Primary Care Units of the Mexican Social Security Institute (IMSS) across 15 States in Mexico (Hidalgo, Sonora, Michoacán, Estado de México, Tamaulipas, Nayarit, Sinaloa, Baja California Sur, Querétaro, Chihuahua, Nuevo León, Puebla, Campeche, Oaxaca, and Mexico City) to evaluate antioxidant enzyme gene variants (*SOD2* rs4880, *GPX7* rs835337, *GPX1* rs1050450, and *CAT* rs1001179). In a subsample of 481 children (237 with normal weight and 244 with obesity) from Campeche, Oaxaca, and Mexico City, serum total antioxidant capacity (sTAC) and oxidative stress markers, including lipid peroxidation (thiobarbituric acid reactive substances, TBARS) and protein carbonyl (PC), were assessed. This protocol was approved by the National Scientific Research Commission and the Ethics Commission of the IMSS (CONBIOETICA-09-CEI-009-20160601; Registration number R-2016-785-100), and it was conducted in accordance with the principles of the Declaration of Helsinki. Informed consent was provided by parents or legal guardians of all participants, and assent was obtained from the children. Children were excluded if they had acute infections, chronic illnesses, were enrolled in a weight reduction program, were taking supplements containing antioxidants, or had fasting plasma glucose levels above 126 mg/dL.

### 2.2. Anthropometric Measurements and Blood Sampling

Body weight was measured using a digital scale (Seca), and height was assessed with a portable stadiometer (Seca). Body mass index (BMI) was computed as weight in kilograms divided by height in meters squared (kg/m^2^). The body weight category was defined according to the BMI percentiles for age and sex proposed by the Centers for Disease Control and Prevention (CDC) (normal weight: BMI 5th–85th percentile; obesity: BMI ≥ 95th percentile) [16]. BMI data were converted into age- and sex-adjusted standard deviation scores (SDS-BMI) using the LMS method following the CDC guidelines [17].

Two blood samples were taken from each participant by venipuncture after at least 8 h of fasting. For genotyping, blood was collected in EDTA tubes, while for the assessment of sTAC and oxidative stress markers, blood was collected in serum separator tubes. Serum was separated via centrifugation and stored at −80 °C until analysis.

### 2.3. DNA Extraction and Genotyping Procedures

Genomic DNA from all participants was isolated from EDTA tubes using the AutoGenFlex STAR (Auto-Gen, Holliston, MA, USA). DNA purity was verified by spectrophotometric analysis at 260/280 nm (BioTek Instruments, Winooski, VT, USA), and DNA integrity was assessed by electrophoresis on 0.8% agarose gels stained with SYBR™ Safe DNA Gel Stain (Thermo Fisher Scientific, Waltham, MA, USA) and visualized under UV light using a Fusion Fx gel documentation system (Vilber Lourmat, Marne-la-Vallee, France). The genotyping of antioxidant enzyme gene variants was performed using TaqMan^®^ SNP Genotyping Assays (Applied Biosystems, Foster City, CA, USA) for *SOD2* rs4880 (C___8709053_10), *GPX7* rs835337 (C_____80748_10), *GPX1* rs1050450 (C_175686987_10), and *CAT* rs1001179 (C__11468118_10) according to the manufacturer’s instructions. Reactions were carried out in 384-well plates using the 7900HT Fast Real-Time PCR system (Applied Biosystems, Foster City, CA, USA), following standard protocols. Genotypes were duplicated in 10% of the sample, and a genotyping concordance rate of 100% was observed.

### 2.4. Serum Total Antioxidant Capacity

To evaluate sTAC levels, we used the spectrophotometric method described by Brand-Williams [18], which involves determining the percentage of 2,2-diphenyl-1-picrylhydrazyl (DPPH) radical scavenging in serum. A 0.1 mM DPPH solution was prepared in 80% methanol (*v*/*v*) and used as the control (A0). Serum samples were diluted 1:30 (*v*/*v*) in DPPH solution (A1), while 80% methanol (*v*/*v*) was considered as the blank (A2). The mixtures were incubated in a dark room for 1 h, followed by centrifugation at 9000× *g* for 10 min at 4 °C. Absorbance of A0, A1, and A2 was measured at 517 nm. The percentage of DPPH radical scavenging was calculated using the formula % DPPH scavenging = (A0 − [A1 − A2]/A0) (100). A standard calibration curve was generated using a 1 mM Trolox stock solution, diluted in DPPH solution to obtain five concentration points ranging from 5 to 50 µM, along with a blank control (Supplementary Table S1). The percentage of DPPH scavenging was calculated for each standard, and the antioxidant capacity of each serum sample was extrapolated from the calibration curve (Supplementary Table S1; Supplementary Figure S1). Results were expressed as millimolar Trolox equivalents (mEq Trolox).

### 2.5. Lipid Peroxidation Assay

Lipid peroxidation in serum samples was quantified using the thiobarbituric acid reactive substances (TBARS) assay, following the methodology described by Jentzsch et al. [19]. A mixture of 300 μL of serum and 200 μL of 0.2 M phosphoric acid was prepared and vortexed thoroughly. Afterward, 25 μL of 0.11 M thiobarbituric acid (TBA) solution was added. The mixture was incubated at 90 °C for 60 min. After incubation, 400 μL of n-butanol was added, followed by vortexing and centrifugation at 15,000× *g* for 10 min at 25 °C. Finally, 200 μL of the supernatant was transferred to a 96-well microplate. Absorbance was measured at 535 nm.

A standard calibration curve was generated using a malondialdehyde (MDA) stock solution prepared from 1,1,3,3-tetramethoxypropane (TMP). Serial dilutions were made to obtain six concentration points ranging from 0.5 to 5 nmol/mL, along with a blank control (Supplementary Table S2; Supplementary Figure S2). Both serum samples and MDA standards were processed under identical conditions. TBARS equivalents were calculated based on the calibration curve, and results were expressed as nanomoles per milliliter (nmol/mL).

### 2.6. Protein Carbonyl Assay

Carbonyl groups were detected by derivatization with 2,4-dinitrophenylhydrazine (DNPH), following the method described by Levine et al. [20]. Two aliquots of each serum sample were used in the assay. One was diluted 1:5 (*v*/*v*) with a DNPH solution (10 mM in 2.5 M HCl), while the other (control) was treated with 2.5 M HCl alone to correct for background absorbance not attributable to DNPH–carbonyl adducts. All tubes were incubated for 1 h at room temperature in the dark. After incubation, 1 mL of 20% trichloroacetic acid solution (TCA) was added, centrifuged at 10,000× *g* for 10 min at 4 °C, and the pellet was washed with 10% TCA under the same conditions, followed by three washes with ethanol/ethyl acetate (1:1, *v*/*v*). Pellets were resuspended in 0.5 mL of 6 M guanidine hydrochloride in 20 mM phosphate buffer (pH 2.5) by vortexing and centrifuged again at 10,000× *g* for 10 min at 4 °C. The absorbance of the supernatant was measured at 370 nm. Carbonyl concentration was calculated using the following equation: protein carbonyl (nmol/mL) = [(CA)/(0.011 μM^−1^)](500 μL/200 μL), where CA is the corrected absorbance (sample—control).

### 2.7. Statistical Analysis

The normal distribution of continuous data was evaluated with the Shapiro–Wilk test, and the rank-based inverse normal transformation was used to transform the variables without normal distribution (Supplementary Table S3). Chi-square and Student’s *t*-test were used to compare frequencies and continuous data between children with normal weight and obesity. All genetic association analyses were conducted under additive, dominant, and recessive inheritance models, considering the variants A for *SOD2* rs4880, A for *GPX7* rs835337, A for *GPX1* rs1050450, and T for *CAT* rs1001179 as effect alleles. Logistic regression models adjusted for age, sex, and location were used to assess the association of genetic variants, sTAC, and oxidative stress markers (TBARS and PC) with obesity. Linear regression analyses adjusted for age, sex, obesity status, and location were performed to analyze the relationship between sTAC and oxidative stress markers (TBARS and PC). Linear regression models adjusted for age, sex, obesity status, and location were employed to evaluate the association between genetic variants, sTAC, and oxidative stress markers (TBARS, PC). To explore whether the genetic variants modulate the relationship between sTAC and oxidative stress markers (TBARS and PC), the participants were stratified into two groups based on their genotype (carriers or non-carriers of the effect allele), according to the inheritance model (additive, dominant, or recessive) that showed a significant association with TBARS or PC in the previous analysis. The association between sTAC and each oxidative stress marker was analyzed separately within each genotype group using linear regression models adjusted for age, sex, obesity status, and location. The statistical analyses were conducted using SPSS software (version 21.0, IBM, Armonk, NY, USA), and two-sided *p*-values < 0.05 were considered statistically significant.

## 3. Results

### 3.1. Characteristics of the Study Population

The general characteristics of the whole study sample are shown in Table 1. A total of 1675 children with normal weight and 1271 children with obesity were included to assess the association of four SNPs, *SOD2* rs4880, *GPX7* rs835337, *GPX1* rs1050450, and *CAT* rs1001179, with obesity. Age showed significant differences between the normal weight and obesity groups (*p* < 0.001). The obesity group showed a higher proportion of boys, as well as higher BMI and BMI z-score values compared to the normal weight group (*p* ≤ 0.009).

We analyzed a subsample to evaluate the relationship between obesity and oxidative stress markers (sTAC: normal weight = 193/obesity = 216; TBARS: normal weight = 237/obesity = 244; PC: normal weight = 227/obesity = 225). The general characteristics of the subsample are described in Supplementary Table S5. Children with obesity showed higher levels of sTAC than children with normal weight (*p* < 0.001). TBARS and PC levels did not present differences between normal weight and obese children (*p* ≥ 0.053).

### 3.2. Association Between Genetic Variants in Antioxidant Enzymes with Childhood Obesity

Table 1 shows the genotype frequencies of the four evaluated SNPs. No statistically significant differences were observed between the normal weight and obesity groups for any SNP (*p* ≥ 0.190). Genotype frequencies were consistent with Hardy–Weinberg Equilibrium (HWE) (*p* ≥ 0.412), except for *CAT* rs1001179 (*p* = 0.030), as shown in Supplementary Table S4. The minor allele frequencies (MAFs) of the SNPs in Mexican children were 33% for *SOD2* rs4880, 23% for *GPX7* rs835337, 12% for *GPX1* rs1050450, and 7% for *CAT* rs1001179. The allelic distributions of *SOD2* rs4880 and *GPX7* rs835337 did not differ from those reported for the adult reference population of individuals of Mexican ancestry living in Los Angeles, according to the 1000 Genomes Project. All MAF values and comparative data are detailed in Supplementary Table S4.

The association of the four SNPs in antioxidant genes with obesity was investigated using an additive, dominant, and recessive model adjusted for sex, age, and location (Table 2). We did not find any significant association between the studied SNPs and childhood obesity (*p* ≥ 0.125).

### 3.3. Association Between Oxidative Stress Markers, Serum Total Antioxidant Capacity, and Obesity

To investigate the relationship between sTAC, TBARS, PC, and obesity, a logistic regression analysis model was performed, adjusting for age, sex, and location (Table 3). The results revealed a significant positive association of sTAC (odds ratio [OR] = 5.58; 95% confidence interval [95% CI]: 1.99–15.5; *p* = 0.001) and TBARS (OR = 1.07; 95% CI: 1.01–1.13; *p* = 0.015) with obesity. No significant association was found for PC (*p* = 0.168, Table 3).

### 3.4. Association Between Serum Total Antioxidant Capacity and Oxidative Stress Markers

To analyze the association between sTAC and oxidative stress markers, a linear regression analysis was performed, adjusting for age, sex, obesity, and location (Table 4). A significant negative association between sTAC and TBARS (β = −2.772 ± 0.765, *p* < 0.001) was observed. No significant association was found for PC (*p* = 0.873, Table 4).

### 3.5. Association Between Genetic Variants in Antioxidant Enzymes, Serum Total Antioxidant Capacity, and Oxidative Stress Markers

The association of the four SNPs with oxidative stress markers and sTAC was assessed through linear regression analysis adjusted for age, sex, obesity, and location under additive, dominant, and recessive models (Table 5). *SOD2* rs4880 was negatively associated with TBARS levels in the recessive model (β = −1.416 ± 0.653, *p* = 0.031). In the additive and dominant models, *GPX1* rs1050450 was negatively associated with TBARS (additive: β = −0.962 ± 0.457, *p* = 0.036; dominant: β = −1.043 ± 0.480, *p* = 0.030) and PC (additive: β = −6.909 ± 2.362, *p* = 0.004; dominant: β = −7.436 ± 2.482, *p* = 0.003). We did not identify any significant association with *GPX7* rs835337 and *CAT* rs1001179 (*p* ≥ 0.068, Table 5).

### 3.6. Effect of SOD2 rs4880 and GPX1 rs1050450 in the Association of Serum Total Antioxidant Capacity with Oxidative Stress Markers

To evaluate whether the SNPs *SOD2* rs4880 and *GPX1* rs1050450 modulate the relationship between sTAC and oxidative stress markers, we assessed these associations separately in non-recessive carriers (GG + GA) and recessive carriers (AA) of *SOD2* rs4880 as well as in non-dominant carriers (GG) and dominant carriers (GA + AA) of *GPX1* rs1050450 (Table 6). A significant negative association between sTAC and TBARS levels was identified in non-recessive carriers (GG + GA) of *SOD2* rs4880 (β = −2.563 ± 0.842, *p* = 0.003). Similarly, a negative association between sTAC and TBARS levels was observed in non-dominant carriers (GG) of *GPX1* rs1050450 (β = −2.899 ± 0.908, *p* = 0.002). In an additional analysis performed in children with normal weight and with obesity separately, the inverse relationship between sTAC and TBARS in non-recessive carriers (GG + GA) of *SOD2* rs4880 (β = −4.564 ± 1.274, *p* < 0.001) and dominant carriers (GA + AA) of *GPX1* (β = −4.961 ± 1.371, *p* < 0.001) remained significant only in children with obesity (Supplementary Table S6).

## 4. Discussion

In the present study, we investigated the direct and modulatory effects of four antioxidant enzyme SNPs (*SOD2* rs4880, *GPX1* rs1050450, *GPX7* rs835337, and *CAT* rs1001179) on the relationships among obesity-related oxidative stress markers in Mexican children. No significant association was identified between SNPs and childhood obesity. However, sTAC and TBARS were positively associated with obesity. Additionally, sTAC was observed in a negative association with TBARS. In the analysis of SNPs, sTAC, and oxidative stress markers, *SOD2* rs4880 was found in negative association with TBARS levels, while *GPX1* rs1050450 was associated with reduced levels of TBARS and PC. Finally, negative associations were identified between sTAC and TBARS in non-recessive carriers (GG + GA) of *SOD2* rs4880 and non-dominant carriers (GG) of *GPX1* rs1050450.

Previous studies have reported associations of SNPs *SOD2* rs4880, *GPX7* rs835337, *GPX1* rs1050450, *CAT* rs1001179 with obesity, body mass index and body fat in Brazilian, Mexican, British, Finnish, Chinese, Caucasian, and African–American adult populations [10,12,13,21,22,23]. However, we did not identify significant associations between these SNPs of antioxidant genes and obesity. This is consistent with the lack of association between *SOD2* rs4880, *GPX1* rs1050450, and obesity reported by Ramírez et al. (2024) in Spanish children [24].

Although it has been documented that increased levels of oxidative stress markers and lower total antioxidant capacity are found in children with obesity [25,26], other studies have not found a significant relationship between TBARS, PC levels, and body weight [27,28]. Our findings show elevated sTAC levels in children with obesity, in agreement with previous studies reporting increased sTAC in pediatric populations with obesity [29,30,31]. Furthermore, an inverse relationship between non-enzymatic antioxidant levels and TBARS has been documented in children with obesity [32,33]. In this context, the inverse association observed between sTAC and TBARS in our study may suggest a compensatory mechanism during obesity development in response to the accumulation of reactive species, the main agents responsible for inducing lipid peroxidation [31,34].

Previous studies have shown that individuals carrying *SOD2* rs4880 A allele exhibit low enzymatic activity of SOD and reduced antioxidant defense [14,35]. However, our results evidence a negative association between *SOD2* rs4880 (recessive model) and TBARS levels, which is consistent with those reported for adults from India [15], where lower lipid peroxidation levels were described among carriers of the T allele of *SOD* rs4880. In the case of *GPX1* rs1050450, while some studies have been inconsistent or reported no association between GPx1 activity, oxidative stress marker levels, and this polymorphism [22,36], a study in women from Poland found lower TBARS levels among A allele carriers of *GPX1* rs1050450 [37]. This is in line with the significant association between the A allele of *GPX1* rs1050450 (dominant model) and reduced levels of TBARS that our results evidence. To our knowledge, there are no previous reports of a significant association between this polymorphism and PC levels in humans. Nevertheless, a *GPX1* knockout mouse model points out the important role of GPx1 in preventing the accumulation of hydroxyl radicals (^•^OH), which are highly reactive and capable of inducing protein oxidation [38]. We did not identify any significant association between oxidative stress markers and the polymorphisms *GPX7* rs835337 and *CAT* rs1001179, which, in the European population, have been linked to lipid peroxidation markers [10,39]. The possible discrepancy between our results and previous reports linking the polymorphisms we studied with deficient antioxidant activity could be related to the complex genetic background of the Mexican population. Specifically, in the Latin American population, it has been described that ancestry and population admixture can modify the effect of genetic variants on health-determining traits [40]. Additionally, it has been shown that loci related to obesity or some of its related traits in the European population do not replicate their effect in Mexican children [41]. In this context, further functional studies and replication in larger and ancestry cohorts are important to better understand the biological mechanisms and potential gene–environment interactions underlying these associations in the Mexican population.

The inverse association between sTAC and TBARS within non-carriers of the variant allele (A) of *SOD2* rs4880 (recessive model) and *GPX1* rs1050450 (dominant model) shows a genotype-dependent association between sTAC and lipid peroxidation. The fact that this association remained significant only in children with obesity suggests a potentially protective role of sTAC against lipid damage in individuals with obesity, who, according to our results, might have compromised antioxidant protection in the absence of the protective genotypes conferred by the A alleles of *SOD2* rs4880 (recessive model) and *GPX1* rs1050450 (dominant model). Our results are consistent with those reported by Hernandez-Guerrero in Mexican adults, in which it was observed that the antioxidant effect (due to a lower level of lipid peroxidation) of a dietary intervention is dependent on the *SOD2* rs4880 genotype [42]. This result highlights the importance of considering genetic variants related to the enzymatic antioxidant system when designing better strategies to prevent or treat complications related to obesity and oxidative stress. The identification of *SOD2 rs4880* AA or *GPX1* rs1050450 GA + AA carriers may support targeted interventions based on diet and physical activity to promote the non-enzymatic antioxidant response and contribute to mitigating oxidative stress and preventing metabolic complications related to obesity.

Among the main strengths of our study, it is important to highlight the inclusion of a large and geographically diverse cohort of Mexican children recruited in 15 states of the country, which provides representativeness and genetic diversity to support our findings. Furthermore, the integrated analysis of genetic variants in antioxidant enzyme genes with sTAC, TBARS, and PC in a well-characterized subgroup provides a more robust exploration of interactions between genetics and oxidative stress. However, our study also presents limitations. As an example, the lack of dietary data prevented the identification of dietary patterns that could influence the redox status. Serum levels of specific antioxidants (such as vitamins C, E, or carotenoids) were not determined, nor was physical activity assessed, both of which may act as additional modulators of oxidative stress. Another important limitation is that we did not measure the enzyme activity of the antioxidant genes evaluated, which would allow us to perform a functional approach to better explain the observed association between polymorphisms, sTAC, and oxidative stress markers. These limitations highlight opportunities for future research aimed at incorporating functional measurements of antioxidant enzymes to establish a possible direct relationship between genotype, enzyme activity, and lifestyle variables in the study of oxidative stress and its genetic modulation in the pediatric population.

## 5. Conclusions

Our results evidence that sTAC and TBARS are inversely related and show positive associations with obesity, suggesting a potential compensatory upregulation of non-enzymatic antioxidant defenses against oxidative stress. Additionally, the inverse relationship between sTAC and TBARS, which only remained significant in non-carriers of the A allele of both *SOD2* rs4880 (recessive model) and *GPX1* rs1050450 (dominant model), highlights the hypothesis that sTAC plays an important protective role in individuals with a possible antioxidant defense compromised by these genetic variants in antioxidant genes.

## Figures and Tables

**Table 1:** Characteristics of the whole study population.

Variable	Normal Weight, *n* = 1675	Obesity, *n* = 1271	*p*-Value
Girls, n (%)	867 (51.8)	596 (46.9)	**0.009**
Boys, n (%)	808 (48.2)	675 (53.1)	
Age, years	9.44 ± 1.96	8.49 ± 1.77	**<0.001**
BMI, kg/m^2^	16.94 ± 3.08	24.56 ±2.79	**<0.001**
BMI z-score	0.14 ± 0.72	1.96 ± 0.65	**<0.001**
sTAC (mEq Trolox) ^(a)^	0.51 ± 0.21	0.59 ± 0.19	**<0.001**
TBARS (nmol/mL) ^(b)^	6.11 ± 3.27	6.74 ± 3.8	0.053
PC (nmol/mL) ^(c)^	41.07 ± 19.03	44.21 ± 16.50	0.061
*SOD2* rs4880			0.531
GG, n (%)	408 (44.1)	399 (46.0)	
GA, n (%)	414 (44.7)	384 (44.2)	
AA, n (%)	104 (11.2)	85(9.8)	
*GPX7* rs835337			0.281
GG, n (%)	469 (57.7)	491 (61.5)	
GA, n (%)	301 (37.0)	267 (33.5)	
AA, n (%)	43 (5.3)	40 (5.0)	
*GPX1* rs1050450			0.868
GG, n (%)	1014 (77.5)	746 (78.2)	
GA, n (%)	280 (21.4)	196 (20.5)	
AA, n (%)	15 (1.1)	12 (1.3)	
*CAT* rs1001179			0.19
CC, n (%)	1148 (87.5)	827 (86.7)	
CT, n (%)	151 (11.5)	123 (12.9)	
TT, n (%)	13 (1.0)	4 (0.4)	

Data are expressed as n (%), mean, and standard deviation. Abbreviations: BMI, body mass index; sTAC, serum total antioxidant capacity; TBARS, thiobarbituric acid reactive substances; PC, protein carbonyl. Analysis by chi-square and Student-t. Significant *p*-values are represented in bold. Sample size analyzed: ^(a)^ Normal weight = 193; Obesity = 216; ^(b)^ Normal weight = 237, Obesity = 244; ^(c)^ Normal weight = 227, Obesity = 225.

**Table 2:** Association between genetic variants in antioxidant enzymes and obesity in children from 15 states in Mexico.

Genetic Variants	*n*	Odds Ratio, Confidence Interval (*p*-Value)		
*SOD2* rs4880	1794	Additive	Dominant	Recessive
		0.93, 0.81–1.08 (0.346)	0.93, 0.77–1.12 (0.464)	0.88, 0.65–1.19 (0.401)
*GPX7* rs835337	1611	Additive	Dominant	Recessive
		0.89, 0.75–1.05 (0.167)	0.85, 0.69–1.05 (0.125)	0.94, 0.60–1.46 (0.766)
*GPX1* rs1050450	2263	Additive	Dominant	Recessive
		0.96, 0.80–1.16 (0.698)	0.95, 0.78–1.17 (0.643)	1.07, 0.50–2.30 (0.867)
*CAT* rs1001179	2266	Additive	Dominant	Recessive
		1.02, 0.81–1.28 (0.887)	1.07, 0.84–1.38 (0.581)	0.42, 0.14–1.31 (0.135)

Data are presented as odds ratio (OR), 95% confidence interval (*p*-value). Analysis by logistic regression model adjusted for age, sex, and location.

**Table 3:** Association of oxidative stress markers and serum total antioxidant capacity with obesity.

Traits	*n*	Odds Ratio (OR), 95% Confidence Interval (*p*-Value)
sTAC (mEq Trolox)	409	5.582, 1.999–15.590 (**0.001**)
TBARS (nmol/mL)	481	1.069, 1.013–1.127 (**0.015**)
PC (nmol/mL)	452	1.007, 0.997–1.018 (0.168)

Data are presented as odds ratio (OR), 95% confidence interval (*p*-value). Abbreviations: sTAC, serum total antioxidant capacity; TBARS, thiobarbituric acid reactive substances; PC, protein carbonyl. Analysis by logistic regression model adjusted for age, sex, and location. Significant *p*-values are represented in bold.

**Table 4:** Association between serum total antioxidant capacity and oxidative stress markers.

Traits	β ± SE (*p*-Value), *n* = 409
TBARS (nmol/mL)	−2.772 ± 0.765 (***p* < 0.001**)
PC (nmol/mL)	−0.734 ± 4.586 (0.873)

Data are presented as β ± standard error (*p*-value). Abbreviations: TBARS, thiobarbituric acid reactive substances; PC, protein carbonyl. Analysis by linear regression model adjusted for age, sex, obesity, and location. Significant *p*-values are represented in bold.

**Table 5:** Association between genetic variants in antioxidant enzymes, serum total antioxidant capacity, and oxidative stress markers.

**Trait**	***SOD2* rs4880**		
	**Additive** ***n* = 421**	**Dominant** ***n* = 218**	**Recessive** ***n* = 33**
sTAC (mEq Trolox)	0.003 ± 0.018 (0.888)	0.004 ± 0.022 (0.866)	0.001 ± 0.043 (0.991)
TBARS (nmol/mL)	0.161 ± 0.279 (0.566)	0.661 ± 0.350 (0.060)	−1.416 ± 0.653 **(0.031)**
PC (nmol/mL)	1.491 ± 1.446 (0.303)	3.098 ± 1.841 (0.093)	−2.206 ± 3.355 (0.511)
**Trait**	***GPX7* rs835337**		
	**Additive** ***n* = 422**	**Dominant** ***n* = 154**	**Recessive** ***n* = 16**
sTAC (mEq Trolox)	−0.036 ± 0.020 (0.068)	−0.044 ±0.023 (0.052)	−0.027 ± 0.060 (0.656)
TBARS (nmol/mL)	0.137 ± 0.311 (0.660)	0.408 ± 0.363 (0.261)	−1.420 ± 0.917 (0.122)
PC (nmol/mL)	1.477 ± 1.631 (0.366)	2.609 ± 1.915 (0.174)	−3.438 ± 4.731 (0.468)
**Trait**	***GPX1* rs1050450**		
	**Additive** ***n* = 434**	**Dominant** ***n* = 69**	**Recessive** ***n* = 2**
sTAC (mEq Trolox)	−0.050 ± 0.030 (0.089)	−0.059 ± 0.031 (0.060)	0.046 ± 0.143 (0.749)
TBARS (nmol/mL)	−0.962 ± 0.457 **(0.036)**	−1.043 ± 0.480 **(0.030)**	−0.475 ± 2.490 (0.849)
PC (nmol/mL)	−6.909 ± 2.362 **(0.004)**	−7.436 ± 2.482 **(0.003)**	−5.306 ± 12.733 (0.677)
**Trait**	***CAT* rs1001179**		
	**Additive** ***n* = 434**	**Dominant** ***n* = 42**	**Recessive** ***n* = 3**
sTAC (mEq Trolox)	0.015 ± 0.037 (0.677)	0.023 ± 0.041 (0.575)	−0.050 ± 0.144 (0.728)
TBARS (nmol/mL)	−0.848 ± 0.540 (0.117)	−0.929 ± 0.581 (0.110)	−0.910 ± 2.489 (0.715)
PC (nmol/mL)	−0.832 ± 2.898 (0.774)	0.341 ± 3.139 (0.914)	−21.773 ± 12.720 (0.088)

Data are presented as β ± standard error (*p*-value). Abbreviations: sTAC, serum total antioxidant capacity; TBARS, thiobarbituric acid reactive substances; PC, protein carbonyl. Analysis by linear regression model adjusted for age, sex, obesity, and location. Significant *p*-values are represented in bold.

**Table 6:** Association between total antioxidant capacity and oxidative stress markers in recessive carriers of *SOD2* rs4880 and dominant carriers of *GPX1* rs1050450.

**Trait**	**Recessive Model *SOD2* rs4880**	
	GG + GA, *n* = 388	AA, *n* = 33
TBARS (nmol/mL)	−2.563 ± 0.842 (**0.003**)	3.634 ± 2.889 (0.226)
PC (nmol/mL)	−3.288 ± 5.165 (0.525)	17.180 ± 25.799 (0.514)
**Trait**	**Dominant Model *GPX1* rs1050450**	
	GG, *n* = 365	GA + AA, *n* = 69
TBARS (nmol/mL)	−2.899 ± 0.908 (**0.002**)	−0.502 ± 1.709 (0.771)
PC (nmol/mL)	−8.614 ± 5.474 (0.117)	6.847 ± 11.998 (0.571)

Data are presented as β ± standard error (*p*-value). Abbreviations: TBARS, thiobarbituric acid reactive substances; PC, protein carbonyl. Analysis by linear regression model adjusted for age, sex, obesity, and location. Significant *p*-values are represented in bold.

## Data Availability

Data from this study are available from the corresponding author upon reasonable request.

## Supplementary Materials

The following supporting information can be downloaded at: https://www.mdpi.com/article/10.3390/antiox14080896/s1, Supplementary Table S1: Trolox standard curve preparation; Supplementary Figure S1: Trolox Standard Curve; Supplementary Table S2: MDA standard curve preparation; Supplementary Figure S2: MDA Standard Curve; Supplementary Table S3: Normality evaluation; Supplementary Table S4: Summary of the quality control of genotyping of genetic variants from our study in Mexican children; Supplementary Table S5: General characteristics and genotypes of the four SNPS in the subsample study population employed to analyze serum total antioxidant capacity and oxidative stress markers, *n* = 481; Supplementary Table S6: Association between total antioxidant capacity and oxidative stress markers in recessive carriers of *SOD2* rs4880 and dominant carriers of *GPX1* rs1050450 separately in children with normal weight and obesity.

## Abbreviations

The following abbreviations are used in this manuscript:

SNPs	Single-nucleotide polymorphisms
sTAC	Serum total antioxidant capacity
PC	Protein carbonyl
TBARS	Thiobarbituric acid reactive substances
TBA	Thiobarbituric acid
OS	Oxidative stress
ROS	Reactive oxygen species
MDA	Malondialdehyde
HNE	4-hydroxy trans-2-nonenal
ONE	4-oxo-2-(E)-nonenal
SOD	Superoxide dismutase
GPx	Glutathione peroxidase
CAT	Catalase
GSH	Reduced glutathione
BMI	Body mass index
DPPH	2,2-diphenyl-1-picrylhydrazyl
DNPH	2,4-dinitrophenylhydrazine
TCA	Trichloroacetic acid
IMSS	Mexican Social Security Institute
TMP	1,1,3,3-tetramethoxypropane
MAF	Minor allele frequencies
