# Supplementary material for: Genetic Variants in Antioxidant Genes Modulate the Relationships Among Obesity-Related Oxidative Stress Markers in Mexican Children
Source: Antioxidants (Basel). 2025 Jul 22;14(8):896. doi: 10.3390/antiox14080896 (PMC12382951; doi:10.3390/antiox14080896)
Supplement: Supplementary file 1 [file antioxidants-14-00896-s001.zip › antioxidants-3723007-supplementary.pdf]

Supplementary Table S1. Trolox standard curve preparation

**Trolox [µMol]**	**% Inhibition**
**0**	0.03
**5**	5.59
**10**	13.24
**20**	28.75
**30**	41.13
**50**	70.99

Supplementary Figure S1. Trolox Standard Curve

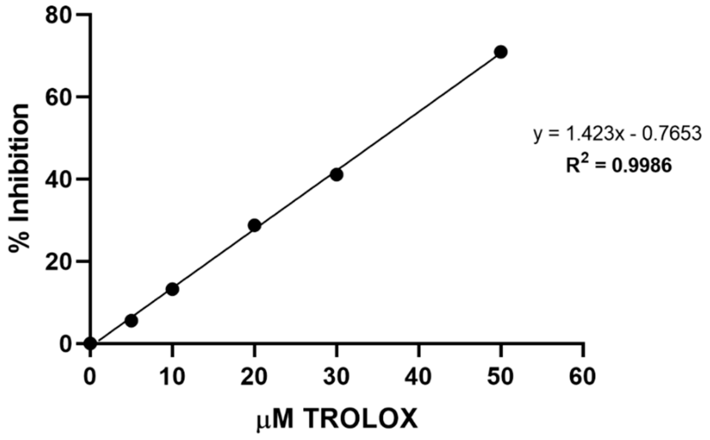

Supplementary Table S2. MDA standard curve preparation

**MDA [nmol/mL]**	**Absorbance**
**0**	0
**0.5**	0.040
**1**	0.079
**2**	0.156
**3**	0.236
**4**	0.311
**5**	0.379

Supplementary Figure S2. MDA Standard Curve

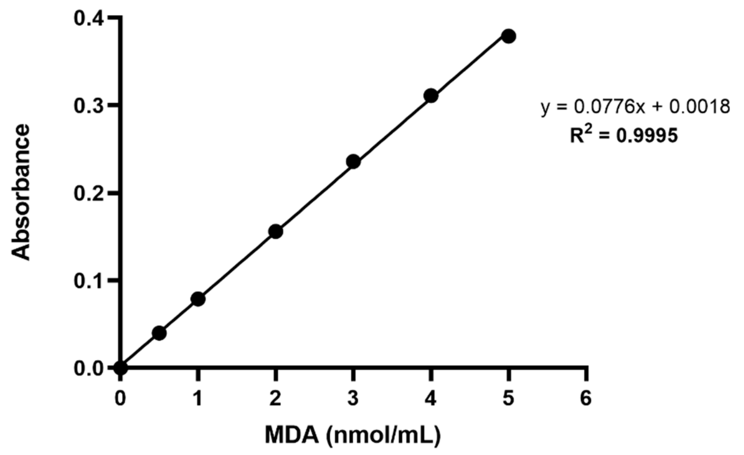

Supplementary Table S3. Normality evaluation.

Trait	Shapiro-Wilk Test (*P*-value)	
	**Untransformed**	**Rank-based inverse normal transformation**
Age, years	**<0.001**	0.999
BMI, kg/m^2	**<0.001**	1.000
BMI z-score	**<0.001**	1.000
sTAC (mEq Trolox)	**<0.001**	1.000
TBARS (nmol/mL)	**<0.001**	1.000
PC (nmol/mL)	**<0.001**	0.995

Shapiro-Wilk tests were performed on metabolic outcomes before and after rank-based inverse normal transformation to determine whether they are normally distributed. Significant *P*-values (<0.05) are represented in bold. Abbreviations: BMI, body mass index; sTAC, serum total antioxidant capacity, TBARS, thiobarbituric acid reactive substances; PC, protein carbonyl.

Supplementary Table S4. Summary of the quality control of genotyping of genetic variants from our study in Mexican children.

Genetic variant	Allelic count Mexican children population				Allelic count 1000 Genome Project			Allele count comparation *p*-value
	Ancestor	Variant	MAF	HWE	Ancestor	Variant	MAF	
SOD2 rs4880 **G/A**	2412	1176	0.327	0.689	83	45	0.352	0.573
GPX7 rs835337 **G/A**	2488	734	0.227	0.931	94	34	0.266	0.318
GPX1 rs1050450 **G/A**	3996	530	0.117	0.412	104	24	0.188	0.015
CAT rs1001179 **C/T**	4224	308	0.067	0.030	108	20	0.156	< 0.001

Chi square test was used to compare the allele counts of our study with the adult Mexican-American reference population from the 1000 Genomes Project. Genotyping quality control included replication of 10% of the samples, with 100% concordance. Abbreviations: MAF, minor allele frequency; HWE, Hardy–Weinberg Equilibrium.

Supplementary Table S5. General characteristics and genotypes of the four SNPS in the subsample study population employed to analyze serum total antioxidant capacity and oxidative stress markers, n= 481

Variable	Normal weight, n = 237	Obesity, n = 244	*P*-value
Girls, n (%)<br>Boys, n (%)	132 (53.2)<br>116 (46.8)	99 (38.8)<br>156 (61.2)	**0.001**
Age, years	9.43 ± 1.72	8.56 ± 1.71	**<0.001**
BMI, kg/m^2	17.16 ± 2.92	24.88 ± 2.95	**<0.001**
BMI z-score	0.26 ± 0.66	2.03 ± 0.66	**<0.001**
sTAC (mEq Trolox) (a)	0.51 ± 0.21	0.59 ± 0.19	**<0.001**
TBARS (nmol/mL)	6.11 ± 3.27	6.74 ± 3.80	0.053
PC (nmol/mL) (b)	41.07 ± 19.03	44.21 ± 16.50	0.610
SOD2 rs4880<br>GG, n (%)<br>GA, n (%)<br>AA, n (%)	99 (47.6)<br>91 (43.8)<br>18 (8.7)	104 (48.8)<br>94 (44.1)<br>15 (7.0)	0.825
GPX7 rs835337<br>GG, n (%)<br>GA, n (%)<br>AA, n (%)	130 (61.9)<br>73 (34.8)<br>7 (3.3)	138 (65.1)<br>65 (30.7)<br>9 (4.2)	0.624
GPX1 rs1050450<br>GG, n (%)<br>GA, n (%)<br>AA, n (%)	177 (81.2)<br>41 (18.8)<br>0 (0)	188 (87.0)<br>26 (12.0)<br>2 (0.9)	0.058
CAT rs1001179<br>CC, n (%)<br>CT, n (%)<br>TT, n (%)	199 (91.3)<br>17 (7.8)<br>2 (0.9)	193 (89.4)<br>22 (10.2)<br>1 (0.5)	0.589

Data are expressed as n (%), mean, and standard deviation. Abbreviations: BMI, body mass index; sTAC, serum total antioxidant capacity; TBARS, thiobarbituric acid reactive substances; PC, protein carbonyl. Analysis by chi-square and Student-t. Significant *p*-values are represented in bold. Sample size analyzed: a) Normal weight = 193; Obesity = 216; b) Normal weight = 227, Obesity = 225.

Supplementary Table S6. Association between total antioxidant capacity and oxidative stress markers in recessive carriers of *SOD2* rs4880 and dominant carriers of *GPX1* rs1050450 separately in children with normal weight and obesity

**Trait**	**Recessive model *SOD2* rs4880**			
	**Normal weight**		**Obesity**	
	GG + GA, n = 190	AA, n = 18	GG + GA, n = 198	AA, n = 15
TBARS (nmol/mL)	-0.586 ± 1.080 (0.600)	0.517 ± 4.990 (0.920)	-4.564 ± 1.274 **(<0.001)**	7.228 ± 3.732 (0.101)
PC (nmol/mL)	-5.177 ± 7.714 (0.503)	-6.777 ± 42.270 (0.877)	-2.083 ± 6.962 (0.765)	21.681 ± 13.337 (0.153)
	**Dominant model *GPX1* rs1050450**			
**Trait**	**Normal weight**		**Obesity**	
	GG, n = 177	GA + AA, n = 41	GG, n = 188	GA + AA, n = 28
TBARS (nmol/mL)	-0.866 ± 1.173 (0.462)	0.202 ± 2.389 (0.933)	-4.961 ± 1.371 **(<0.001)**	-2.512 ± 2.219 (0.273)
PC (nmol/mL)	-11.386 ± 8.336 (0.174)	9.294 ± 18.689 (0.624)	-5.849 ± 7.293 (0.424)	6.220 ± 11.926 (0.609)

Data are presented as β ± standard error (p-value). Abbreviations: TBARS, thiobarbituric acid re-active substances; PC, protein carbonyl. Analysis by linear regression model adjusted for age, sex, obesity, and location. Significant p-values are represented in bold.
